# Highly differentiated cellular and circuit properties of infralimbic pyramidal neurons projecting to the periaqueductal gray and amygdala

**DOI:** 10.3389/fncel.2015.00161

**Published:** 2015-04-28

**Authors:** Ashley N. Ferreira, Hanna Yousuf, Sarah Dalton, Patrick L. Sheets

**Affiliations:** ^1^Department of Biological Sciences, University of Notre DameNotre Dame, IN, USA; ^2^Department of Pharmacology and Toxicology, Indiana University School of Medicine-South BendSouth Bend, IN, USA

**Keywords:** infralimbic cortex, cortico-PAG neurons, cortico-amygdalar neurons, retrograde labeling, slice electrophysiology, circuit mapping, periaqueductal gray, basolateral amygdala

## Abstract

The infralimbic (IL) cortex is a key node in an inter-connected network involved in fear and emotion processing. The cellular and circuit-level mechanisms whereby IL neurons receive, filter, and modulate incoming signals they project onward to diverse downstream nodes in this complex network remain poorly understood. Using the mouse as our model, we applied anatomical labeling strategies, brain slice electrophysiology, and focal activation of caged glutamate via laser scanning photostimulation (glu-LSPS) for quantitative neurophysiological analysis of projectionally defined neurons in IL. Injection of retrograde tracers into the periaqueductal gray (PAG) and basolateral amygdala (BLA) was used to identify cortico-PAG (CP) and cortico-BLA (CA) neurons in IL. CP neurons were found exclusively in layer 5 (L5) of IL whereas CA neurons were detected throughout layer 2, 3, and 5 of IL. We also identified a small percentage of IL neurons that project to both the PAG and the BLA. We found that L5 CP neurons have a more extensive dendritic structure compared to L5 CA neurons. Neurophysiological recordings performed on retrogradely labeled neurons in acute brain slice showed that CP and CA neurons in IL could be broadly classified in two groups: neuronal resonators and non-resonators. Layer 2 CA neurons were the only class that was exclusively non-resonating. CP, CA, and CP/CA neurons in layers 3 and 5 of IL consisted of heterogeneous populations of resonators and non-resonators showing that projection target is not an exclusive predictor of intrinsic physiology. Circuit mapping using glu-LSPS revealed that the strength and organization of local excitatory and inhibitory inputs were stronger to CP compared to CA neurons in IL. Together, our results establish an organizational scheme linking cellular neurophysiology with microcircuit parameters of defined neuronal subclasses in IL that send descending commands to subcortical structures involved in fear behavior.

## Introduction

The medial prefrontal cortex (mPFC) is critical for processing emotional responses. Output from the mPFC is essential in the expression and extinction of conditioned fear (Morgan et al., 1993; Morgan and LeDoux, 1999; Corcoran and Quirk, 2007; Laurent and Westbrook, 2008; Sotres-Bayon and Quirk, 2010; Cho et al., 2013). Decreased mPFC activity is associated with fear extinction deficits (Morgan et al., 1993), and is a key feature of post-traumatic stress disorder (Shin et al., 2004; Gold et al., 2011). L5 pyramidal neurons are the major subcerebral output pathway of mPFC. Across the cortex, including mPFC, L5 contains a heterogeneous population of pyramidal neurons that have distinct local circuit organization, firing properties, interconnectivity, morphology, and neuromodulation based on long-range projection target (Molnar and Cheung, 2006; Morishima and Kawaguchi, 2006; Wang et al., 2006; Le Be et al., 2007; Brown and Hestrin, 2009; Anderson et al., 2010; Dembrow et al., 2010; Morishima et al., 2011; Sheets et al., 2011; Kiritani et al., 2012; Oswald et al., 2013). Thus, functional characteristics of L5 pyramidal neurons within mPFC subregions, including the IL, are likely to vary. Behaviorally, the IL is a key component in the acquisition and recall of fear extinction (Sierra-Mercado et al., 2006; Vidal-Gonzalez et al., 2006; Sierra-Mercado et al., 2011). However, the specific dynamics and circuit organization of neurons comprising descending IL output that contributes to fear perception and the corresponding conditioned response remains unclear.

Tracer studies in monkey (Mantyh, 1982; An et al., 1998), cat (Mantyh, 1982), and rat (Hardy and Leichnetz, 1981; Beitz, 1982; Mantyh, 1982; Herrero et al., 1991; Shipley et al., 1991; Floyd et al., 2000; Gabbott et al., 2005) demonstrated that the mPFC sends projections to the PAG, a midbrain structure that integrates motivational/limbic and sensory input to initiate specific outputs such as threat-coping behavior (Bandler and Carrive, 1988; Bandler and Depaulis, 1988). The mPFC also projects to the amygdala (Hurley et al., 1991; Brinley-Reed et al., 1995; McDonald, 1998; Quirk et al., 2003; Vertes, 2004; Hubner et al., 2014), a structure pivotal in fear acquisition and fear extinction (see review, Davidson, 2002). Top–down regulation of the amygdala by the mPFC is well-known (Quirk et al., 2003; Likhtik et al., 2005, 2014). Additionally, there are reciprocal pathways between the PAG and amygdala (Hopkins and Holstege, 1978; Rizvi et al., 1991), which are critical in pain (Behbehani, 1995) and fear conditioning (Johansen et al., 2010; McNally et al., 2011; Kim et al., 2013; Penzo et al., 2014). Inactivating the PAG reduces amygdalar response to aversive stimuli and attenuates fear learning (Johansen et al., 2010). Circuit-based models of anxiety disorders suggest that abnormal activity in amygdala and mPFC mediates inadequate regulation of fear responses (Greenberg et al., 2012).

Together, these findings indicate that coordination between the mPFC, PAG, and amygdala is essential for normal fear behavior. However, cellular and circuit physiology of specific populations of mPFC neurons targeting the PAG and amygdala is unknown but essential in understanding the neurobiology underlying fear processing and anxiety disorders. Here we identified and characterized populations of IL neurons that send axonal projections to the PAG, to the basolateral amygdala (BLA), and to both. We show that these neurons constitute projection classes with distinct morphology and local cortical circuits, but heterogeneous intrinsic physiology. Our findings provide a framework for delineating the functionality of IL neurons in signaling PAG-BLA networks associated with fear learning.

## Materials and Methods

### Animals

Wild-type C57Bl/6J mice (Jackson Laboratories; total *n* = 104) were used in accordance with the animal care and use guidelines of Indiana University, National Institutes of Health, and Society for Neuroscience. Animal experiments were approved by the Institutional Animal Care and Use Committee (IACUC) at both Indiana University and the University of Notre Dame.

### Retrograde Labeling

At postnatal days 21–24, mice (~8–10 g) of either gender underwent injection of retrograde tracer into the PAG or BLA as described below. Retrograde tracers were either cholera toxin β-subunits conjugated with Alexa dyes or fluorescent retrobeads (Lumafluor). Mice were anesthetized with 1.5% isoflurane in 100% O_2_ with a flow rate of 0.6 L/min (SurgiVet Isotech 4, Smith). Body temperature was maintained at 37°C using a feedback-controlled heating pad (FHC). The head was stabilized in a stereotaxic frame (900 series, Kopf Instruments). Pipettes for injections were fabricated from calibrated micropipettes (Wiretrol II, 5-000-2010, Drummond) using a vertical puller (Model PP-830, Narishige). Before surgery, buprenorphine HCl (0.03 mg/kg) was injected subcutaneously for pain relief. For PAG and BLA injections, the scalp was incised, a craniotomy was made, the dura was reflected, and pipettes were advanced to reach the stereotaxic coordinates of the desired target. The pipette was advanced to the intracranial target and submicroliter volumes (~100–200 nL) of retrograde tracer were injected using a Nanoject II (Stoelting). The pipette was kept in place for at least 60 s to limit tracer reflux out of the injection site. Following surgery, meloxicam (0.25 mg/kg) was injected subcutaneously for pain relief during recovery. Animals were allowed to recover for at least 48 h to ensure robust retrograde labeling.

### Stereotaxic Coordinates

For PAG injections, the head was fixed at a 30° down angle. Coordinates for PAG injections were (relative to lambda): 2.8 mm caudal, 0.5 mm lateral, and 2.5 mm deep also at a 52° angle off the horizontal plane. The small size of the mouse PAG limited our ability to effectively target locations along the anterior–posterior (AP) axis. Therefore, while injections could be targeted along the dorsal-ventral (DV) axis of the PAG they spread to multiple locations along the AP axis. For BLA injections, coordinates were (relative to bregma): 1.1 caudal, 3.4 mm lateral, and 3.9 mm deep at a 4° angle off the vertical plane.

### Slice Preparation

Acute brain slice experiments were performed using mice of either gender at a postnatal age of 23–32 days. Brain slices were prepared as described (Anderson et al., 2010; Sheets et al., 2011) at postnatal days 23–32 (i.e., 2–8 days after bead injections). Modified coronal slices (spine of the blade tilted rostrally 10–20°; 300 μm thick) containing the IL were made by vibratome-sectioning the brain (VT1200S, Leica) in chilled cutting solution (composed of, in mM: 110 choline chloride, 25 NaHCO_3_, 25 D-glucose, 11.6 sodium ascorbate, 7 MgSO_4_, 3.1 sodium pyruvate, 2.5 KCl, 1.25 NaH_2_PO_4_, and 0.5 CaCl_2_). The AP range for slices was approximately +1.8 to +0.3 mm relative to bregma. Slices were transferred to artificial cerebrospinal fluid (ACSF, composed of, in mM: 127 NaCl, 25 NaHCO_3_, 25 D-glucose, 2.5 KCl, 1 MgCl_2_, 2 CaCl_2_, and 1.25 NaH_2_PO_4_, aerated with 95% O_2_/5% CO_2_) at 37°C for 30 min. Slices were subsequently incubated in ACSF at 22°C for at least 1 h prior to electrophysiological recordings.

### Laminar Boundaries

Previously described methods in the motor cortex (Suter et al., 2013) were used to identify laminar boundaries in IL. Acute brain slices were visualized at 4x magnification using differential interference contrast (DIC) optics with infrared illumination. Digital images at this magnification were acquired and analyzed off-line. Laminar boundaries in IL could easily be identified using this imaging method. The pia was easily identifiable at the midline of the coronal slice. Layer 1 (L1) contained no cell bodies. L2 was narrow and optically dense followed by a lighter and equally narrow L3. L5 was also optically dense with larger cell bodies. The mean thickness (±SD) of IL measured in 300 μm coronal slices (range relative to bregma: +1.8 mm to +0.3 mm) was 252 ± 43 μm. Measurements of laminar boundaries in IL (*n* = 62) normalized to the full thickness of IL (pia = 0; white matter = 1) were consistent. The mean normalized depths of laminar borders (±SD) were L1/2, 0.16 ± 0.03; L2/3, 0.25 ± 0.05; and L3/5, 0.35 ± 0.06. The border distinguishing IL from the prelimbic cortex (PL) was identified by both a distinguishable narrowing of L3 and an infiltration of L2 cells into L1 in IL (Van De Werd et al., 2010).

### Fluorescence Imaging and Analysis

Acute cortical slices were visualized under LED optics (coolLED) and fluorescence intensity analyses were performed using custom routines in Matlab (Mathworks). Images were rotated to align the pia horizontally and regions of interest spanning the entire cortical thickness and containing labeled neurons in IL were selected. The pixel intensities (0–256) in these regions of interest were averaged along the rows, yielding a profile representing the average pixel intensities along the medial-lateral axis, showing the radial distribution of fluorescence in the IL. Next, we performed background subtraction to reduce the autofluorescence signal by fitting a polynomial to the non-fluorescent portions of the profile and subtracting a calculated background profile from the raw profile. Following background subtraction, normalized fluorescence intensity was determined by dividing all pixel values by the maximum pixel intensity.

### Electrophysiology

Whole-cell recordings from fluorescently labeled pyramidal neurons were performed as described (Sheets et al., 2011). Briefly, slices were transferred to the recording chamber of a SliceScope Pro 6000 (Scientifica) containing an upright microscope (BX51, Olympus) and PatchStar micromanipulators (Scientifica). Brain slices were held in place with short pieces of flattened gold wire (0.813 mm diameter; Alfa Aesar). We targeted fluorescently labeled neurons that were visualized using LED optics (coolLED). CP and CA neurons were identified by fluorescence of red or green retrobeads or cholera toxin β-subunits conjugated with Alexa dyes. Pipettes for recordings were fabricated from borosilicate capillaries with filaments (G150-F, Warner) using a horizontal puller (P-97, Sutter), and filled with intracellular solution composed of (in mM) 128 K-gluconate, 10 HEPES, 1 EGTA, 4 MgCl_2_, 4 ATP, and 0.4 GTP, 10 phosphocreatine, 3 ascorbate, and 0.05 Alexa-594 or 488 (Molecular Probes); pH 7.3. EGTA was included both to facilitate seal formation and to reduce cytosolic calcium elevations induced by the various stimulus protocols used in these studies. ACSF was used as the extracellular recording solution. Biocytin (2–3 mg; Sigma) was also added for subsequent two-photon imaging of recorded neurons. Slices were ideally used 1.5–3 h after preparation, but some were used up to 6 h after preparation. Recordings were performed at 22°C or 34°C as noted. ACSF was refreshed every 3 h at 22°C and every 2 h at 34°C. The recording temperature was controlled by an in-line heating system (TC324B, Warner). Recordings were targeted to neurons 60–100 μm deep in the slice. Intrinsic recordings (34°C) were performed with synaptic blockers (in μM): 5 CPP, 10 NBQX, and 5 GABAzine. Pipette capacitance was compensated; series resistance was monitored but not compensated, and required to be <35 MΩ for inclusion in the data set. Current-clamp recordings were bridge-balanced. Current was injected as needed to maintain the membrane potential near -70 mV during select stimulus protocols (i.e., within the activation range of *I*_h_ at baseline). Recordings were amplified and filtered at 4 kHz and digitized at 10 kHz using a Multiclamp 700B amplifier (Molecular Devices). Membrane potential values were not corrected for a calculated liquid junction potential of 10 mV (22°C) or 11 mV (34°C).

Voltage sag and resonant frequency were measured from a membrane potential of -70 mV. Voltage sag was measured by presenting multiple 1 s hyperpolarizing current steps (-200 pA, -150 pA, -100 pA, -50 pA). Percentage voltage sag was calculated from the peak voltage (V_peak_) and steady-state voltage (V_steady-state_), as 100 × (*V*_peak_ – *V*_steady-state_)/*V*_peak_ for traces where V_steady-state_ was similar between groups. A chirp stimulus consisting of a sinusoidal current of constant amplitude [adapted from (Shin et al., 2008; Sheets et al., 2011)] was adjusted to produce an ~5 mV response at the maximum frequency of 20 Hz. Responses to chirp stimuli (20 s duration) were analyzed by calculating impedance as the magnitude of the ratio of the Fourier transform of the responses (in mV) to the Fourier transform of the stimulus (in pA). This analysis was used to obtain impedance amplitude profiles (ZAPs). Resonant frequencies were identified from ZAPs as the peak in the frequency-domain (0.5-20 Hz; boxcar smoothed with a 0.75 Hz window).

### Glutamate Uncaging and Laser Scanning Photostimulation

Glutamate uncaging and laser scanning photostimulation were performed as described previously (Weiler et al., 2008; Anderson et al., 2010), using an ultraviolet (UV) laser (355 nm; DPSS Lasers, Inc.). *Ephus* software was used for hardware control and data acquisition (http://www.ephus.org; Suter et al., 2010). The bath solution for photostimulation studies contained elevated concentrations of divalent cations (4 mM Ca^2+^ and 4 mM Mg^2+^) and a NMDA receptor antagonist (5 μM CPP; Tocris) to dampen neuronal excitability. Caged glutamate (0.2 mM; MNI-glutamate, Tocris) was added to a recirculating ACSF bath solution. Voltages were not corrected for liquid junction potential. Recordings were performed at 21°C and were monitored for series resistance (inclusion criterion: <40 MΩ; mean: ~25 MΩ). Once a patch recording of a labeled neuron was established, an image of the slice (4x objective) was acquired before mapping. This image was used for precise registration of the mapped grid. The mapping grid (16 × 16; 75 μm spacing) was rotated with the top row of the grid flush with the pia and the soma was centered horizontally in the grid. The grid locations were sampled (every 0.4 s) with a UV stimulus 1.0 ms in duration and 20 mW at the specimen plane. For excitatory recordings, patch pipettes contained potassium-based intracellular solution (in mM: 128 K-gluconate, 10 HEPES, 1 EGTA, 4 MgCl_2_, 4 ATP, and 0.4 GTP, 10 phosphocreatine, 3 ascorbate, and 0.05 Alexa-594 or 488 hydrazide). For inhibitory recordings, equimolar cesium was substituted for potassium, and 1 mM QX-314 was added to the intracellular solution. Photostimulation sites resulting in activation of glutamate receptors in the membrane of the recorded neuron were readily detected based on characteristically short onset latencies (<7 ms) of responses (Schubert et al., 2001; Anderson et al., 2010), and excluded from analysis. All remaining recorded inputs with onset latencies greater than 7 ms were included in the map analysis as synaptic responses resulting from uncaged glutamate activation of presynaptic neurons within the local circuit. Excitatory (glutamatergic) responses were recorded at a command voltage of -70 mV. Excitatory input maps were constructed on the basis of the mean inward current over a 0–50 ms post-stimulus time window. Inhibitory (GABAergic) responses were recorded at a command voltage of +10 mV. Inhibitory input maps were constructed on the basis of the mean outward current over a 0–750 ms post-stimulus time window. The inhibitory post-stimulus time window was increased due to the longer time course for inhibitory responses to return to baseline. Animal numbers reported for inhibitory maps are a subset of animals reported for excitatory maps because excitatory and inhibitory maps were acquired in the same recorded neuron. In some cases, following acquisition of excitatory input maps, the recording was lost and subsequent inhibitory maps could not be acquired.

### Confocal Imaging

Following retrograde tracer injections (at least 48 h), brains were fixed by cardiac perfusion with fixative (4% paraformaldehyde in PBS). Brain sections containing IL were cut by vibratome at a thickness of 50 μm. Confocal fluorescent images of retrogradely labeled CP and CA neurons in IL were obtained using a Zeiss LSM 710 confocal microscope equipped with an argon (488 nm) and diode (561 nm) laser.

### Two-Photon Imaging and Morphology

Following recording, neurons were quickly examined under epifluorescence microscopy to confirm the preservation of basal and apical dendrites. Slice processing and preparation for imaging was performed as previously described (Suter et al., 2013). Biocytin-filled neurons were imaged using an Olympus FV1000 multiphoton microscope equipped with an Olympus 25X water objective. Images were stitched, reconstructed, and analyzed using Neurolucida software (MBF Bioscience). Both the Zeiss and Olympus microscopes are part of the Imaging and Flow Cytometry Core Facility at Indiana University School of Medicine-South Bend.

### Pharmacology

*I*_h_ was blocked using low concentrations of the irreversible HCN channel blocker, ZD7288 (Tocris). A 25 mM stock solution was made in water, aliquoted, and stored at –20°C. Aliquots were diluted in ACSF for experiments to a final concentration of 10 μM. Stock solutions of MNI-caged glutamate (50 mM in water) were prepared at room temperature (to avoid precipitation), sonicated and stored in 30 μL aliquots at -20°C until use.

### Statistical Analysis

For all data, a Lilliefors test was performed prior to significance testing to determine if the data were normally distributed. Significance between multiple (i.e., 3-4) independent groups was determined using a one-way ANOVA for normally distributed data or a Kruskal–Wallis test for non-normally distributed data. A Bonferroni *post hoc* analysis for multiple comparisons was subsequently conducted if the one-way ANOVA or Kruskal–Willis test resulted in a significant omnibus *F*-test. Unpaired comparisons between two independent groups were performed with the Student’s unpaired *t*-test (for normally distributed data) or the Wilcoxin rank sum test (for non-normally distributed data). Error bars in plots represent standard error of the mean (SEM).

## Results

### Laminar Distributions of CP and CA Neurons in IL

**FIGURE 1:**
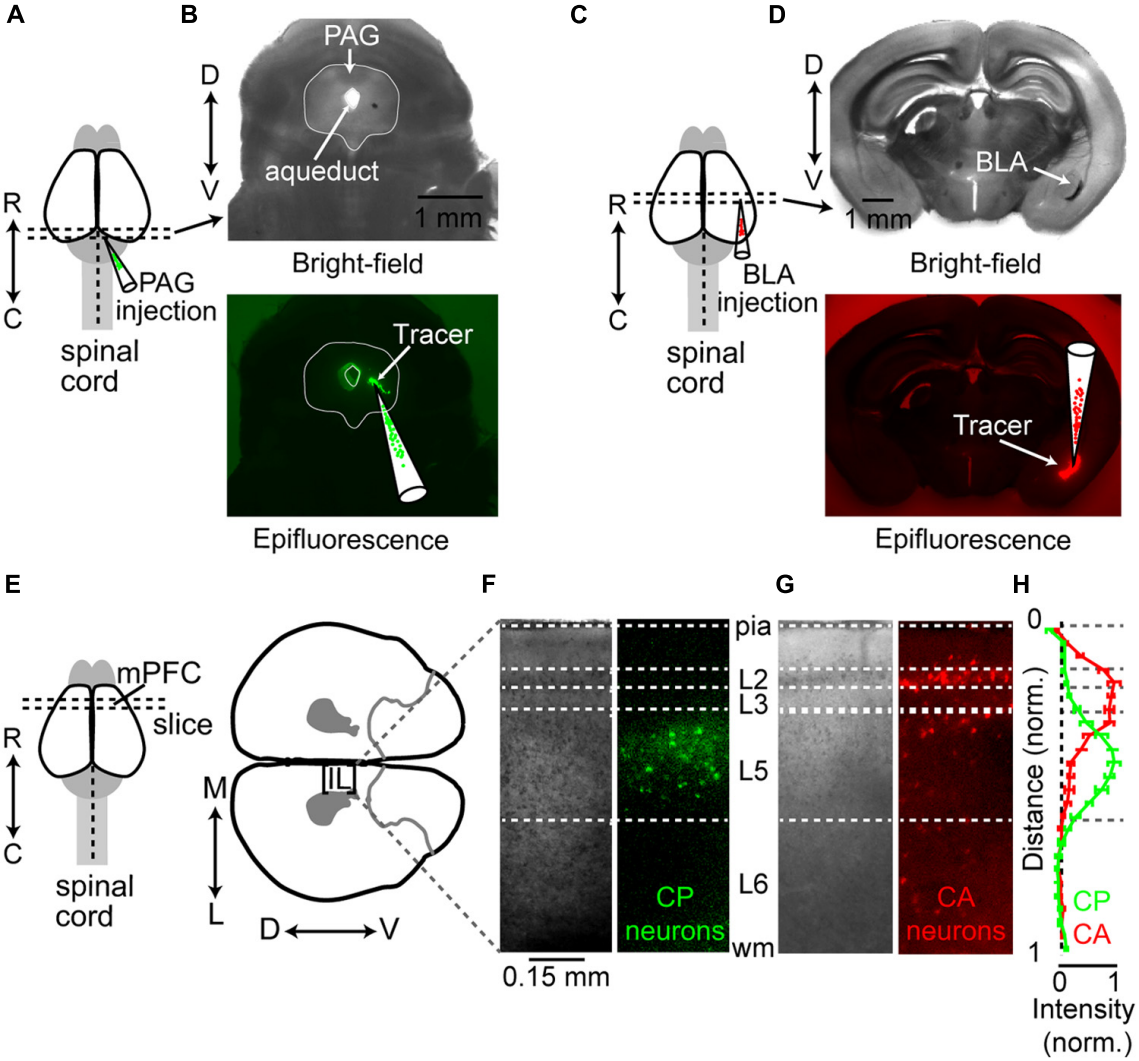
**Differential distribution of CP and CA neurons in IL. (A)** Schematic depicting the injection of fluorescent retrograde tracer into the PAG. Injection locations in the PAG were confirmed in coronal slices (300 μm) visualized using both bright-field (**B**, top) and epifluorescence (**B**, bottom) imaging. **(C)** Schematic depicting the injection of retrograde fluorescent tracer injection into the basolateral amygdala (BLA). **(D)** Bright-field image (top) of coronal brain slice (300 μm) containing the BLA and the corresponding epifluorescence image (bottom) confirming the location of retrograde tracer injection. **(E)** At least 2 days following tracer injection, coronal slices (300 μm) containing the mPFC were prepared (left) and oriented so that the pia of the IL cortex was horizontal in the imaging chamber (right). Bright-field and epifluorescence images showing laminar location of retrogradely labeled **(F)** CP neurons and **(G)** CA neurons in IL cortex. **(H)** Normalized fluorescence intensity (mean ± SEM) of soma location as a function of normalized cortical distance [where pia = 0 and white matter (wm) = 1] for CP (*n* = 10) and CA (*n* = 7) neurons in IL cortex. Rostral-caudal: R↔C; dorsal-ventral: D↔V; lateral-medial: L↔M.

**FIGURE 2:**
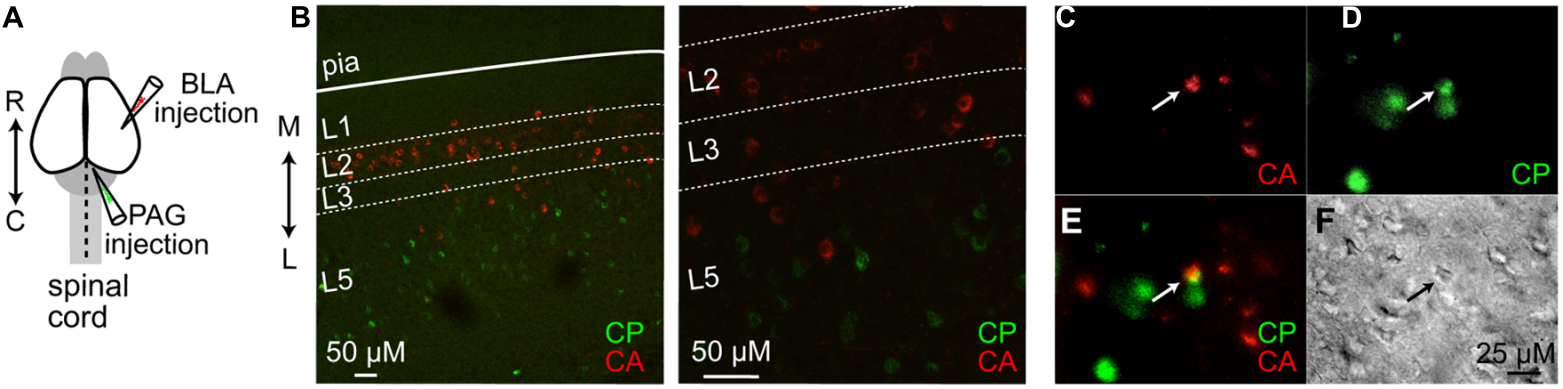
**Identifying double-labeled CP/CA neurons in IL. (A)** Schematic depicting the concurrent injection of spectrally distinct retrograde fluorescent tracers into the BLA and PAG. **(B)** Confocal images at 10x and 25x magnification of retrogradely labeled CP (green) and CA (red) neurons in the IL cortex in a 50 μm coronal slice. Epifluorescent images at 60x magnification showing fluorescently labeled **(C)** CA neurons, **(D)** CP neurons, and the corresponding **(E)** overlay and **(F)** bright-field image. Arrows in each image identify the double-labeled CP/CA neuron. Rostral-caudal: R↔C; lateral-medial: L↔M.

To identify and target projectionally defined neurons in IL for electrophysiological recording and circuit mapping, we injected fluorescent retrograde tracers into the PAG (**Figures 1A,B**) or BLA (**Figures 1C,D**) At least 2 days following retrograde tracer injection, we prepared coronal brain slices of the mPFC that included IL (**Figure 1E**). Labeled CP neurons were distributed in L5 of ipsilateral IL (**Figures 1F,H**). This is consistent with older tracer studies showing that the mPFC sends axonal projections to the PAG (Shipley et al., 1991). Injections of retrograde tracer into BLA resulted in labeled CA neurons distributed in L2 of ipsilateral IL (**Figures 1G,H**), consistent with previous findings (Gabbott et al., 2005; Hirai et al., 2012; Little and Carter, 2013). However, we also detected retrograde labeling of CA neurons in both L3 and L5 of ipsilateral IL (**Figures 1G,H**). Previous studies in the rat have shown that a small percentage of IL neurons project to both the BLA and PAG (Gabbott et al., 2005). To test if this was consistent in mice, we performed concurrent injections of spectrally distinct retrograde tracers (cholera toxin β-subunits conjugated with Alexa dyes) in the PAG and BLA (**Figure 2A**). We found that CP and CA neurons in L5 of IL were in close proximity (**Figure 2B**) and that a small percentage of IL neurons were labeled with both retrograde tracers (**Figure 2C**) These results show IL output pathways to the PAG and BLA consist primarily of neighboring populations neurons in L2–L5 of IL, but with a small subset of L5 neurons projecting to both subcortical structures.

### CP and L5 CA Neurons in IL Differ Morphologically

**FIGURE 3:**
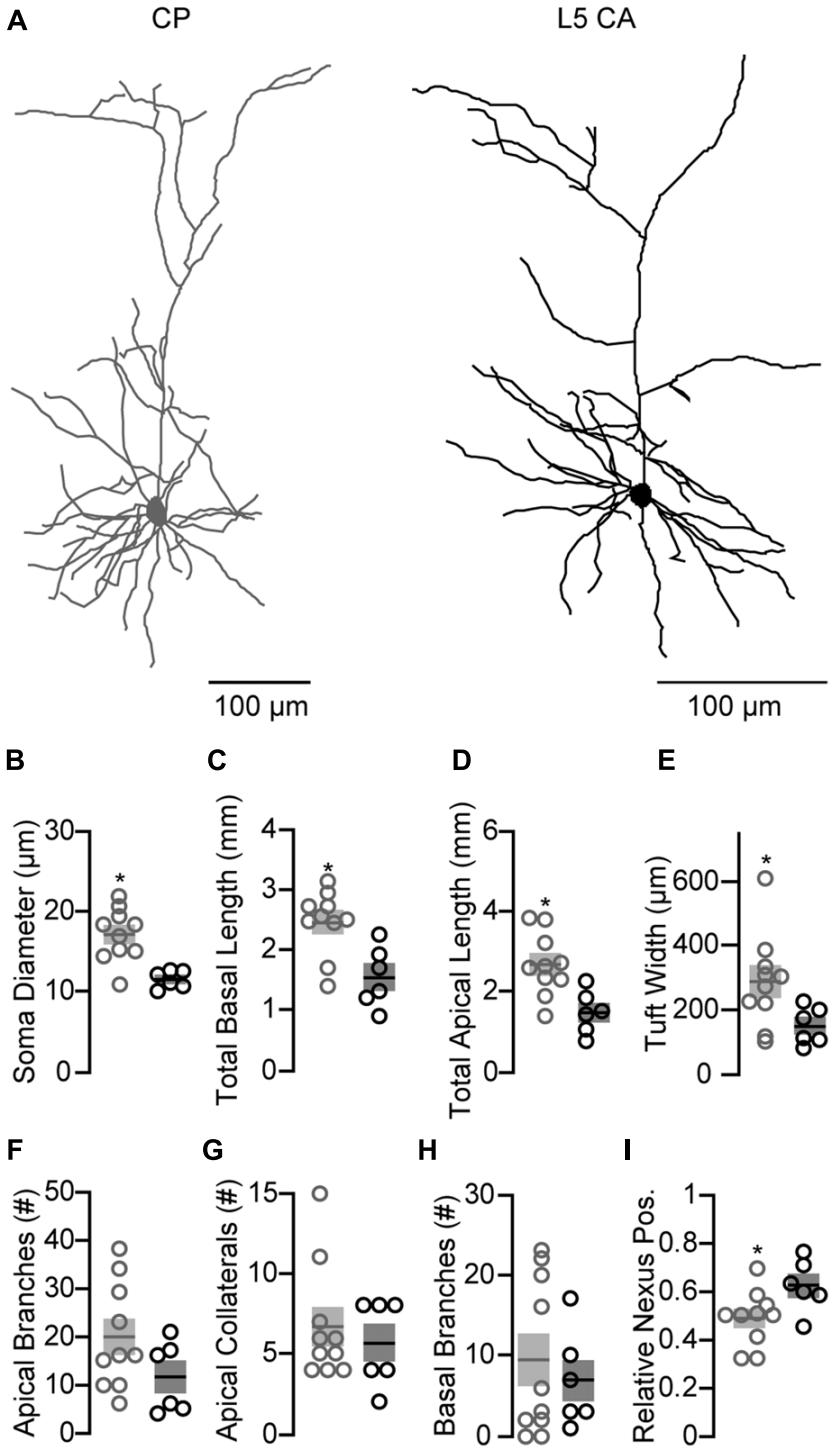
**Morphological disparities between CP and CA neurons in L5 of IL. (A)** Representative morphological reconstructions of CP and CA neurons in L5 of IL. **(B–I)** Mean (±SEM) morphological properties for the two projection classes. (^∗^p < 0.05, Student’s unpaired *t*-test).

In rat frontal cortex, L5 neurons differ morphologically based on projection target (Morishima and Kawaguchi, 2006; Otsuka and Kawaguchi, 2008; Dembrow et al., 2010; Hirai et al., 2012). We tested if this was also true in mouse IL by adding Biocytin to the intracellular recording solution which allowed for morphological assessment of recorded neurons using immunohistochemistry and two-photon imaging (see Materials and Methods). Neuronal reconstructions revealed morphological differences between CP (*n* = 10) and L5 CA (*n* = 6) neurons (**Figure 3A**). Compared to L5 CA neurons, CP neurons had significantly larger somas and greater basal dendrite length, apical dendrite length, and apical tuft width (**Figures 3B–D**). However, the number of apical branch points, basal branch points, and apical collaterals did not differ significantly (**Figures 3E–H**). Analysis of relative nexus (major branch point) position on the apical dendrite shows that CP neurons have a deeper apical bifurcation compared to L5 CA neurons (**Figure 3I**). Unique morphology between these neuronal classes suggests significant differences in their dendritic integration, cellular excitation, and function within IL cortical circuits.

### Projection Target and Cortical Layer Specificity Determines Intrinsic Properties of CP and CA Neurons in IL

**FIGURE 4:**
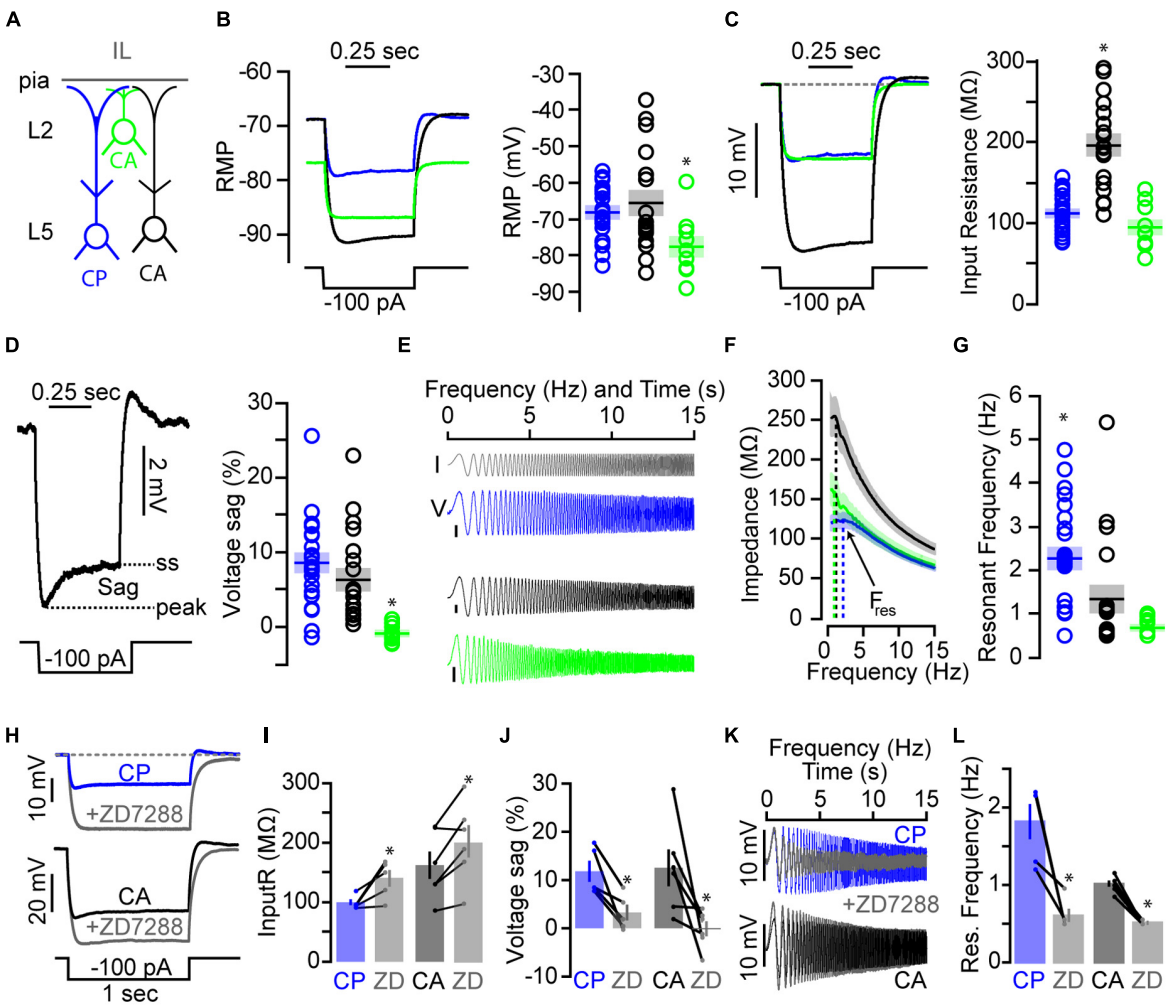
**Subthreshold physiology differs between CP and CA neurons in IL **(A)** Schematic showing the subclasses of neurons recorded (CP, cortico-PAG; CA, cortico-BLA; L2, layer 2; L5, layer 5).** (**B**, left) Average traces of subthreshold responses for each neuronal subclass. (**B**, right) Mean (±SEM) resting membrane potential (RMP) for CP (*n* = 26), L3/5 CA (*n* = 20), and L2 CA (*n* = 10) neurons (^∗^*p* < 0.05, Kruskal–Wallis test). (**C**, left) Subthreshold traces aligned to RMP showing the differences in input resistance. (**C**, right) Mean (±SEM) input resistance across projection class (^∗^*p* < 0.05, one-way ANOVA). (**D**, left) Subthreshold voltage response displaying a sag in the membrane potential before reaching a steady state (ss) level. (**D**, right) Mean (±SEM) voltage sag % across projection class (^∗^*p* < 0.05, Kruskal–Wallis test). **(E)** Chirp current **(I)** stimulus (gray) and examples of chirp voltage (V) responses for CP, L3/5 CA, and L2 CA neurons. Only the first 15 s of the sweeps are shown. Scale bars = 5 mV. **(F)** Average impedance profiles (ZAPs) with dashed lines denoting resonant frequency (*F*_res_). **(G)** Mean (±SEM) resonant frequency across projection class (^∗^*p* < 0.05, Kruskal–Wallis test). **(H)** Subthreshold response of a CP (top) and L3/5 CA (bottom) neuron before and after addition of the *I*_h_-blocker ZD7288 (10 μM). Effects of ZD7288 on **(I)** input resistance, **(J)** voltage sag, and **(K,L)** resonant frequency of CP (*n* = 5) and L3/5 CA (*n* = 6) neurons.

Pyramidal neurons in the cortex are often broadly categorized as either pyramidal tract (PT) or intratelencephalic (IT; Molyneaux et al., 2007; Reiner, 2010; Shepherd, 2013; Dembrow and Johnston, 2014). Axons of PT neurons project to targets in the brainstem and spinal cord, whereas IT axons remain entirely IT and project bilaterally via the corpus callosum. Multiple studies show that intrinsic and circuit profiles of neocortical pyramidal neurons differ based on their PT-IT classification (Kasper et al., 1994; Molnar and Cheung, 2006; Hattox and Nelson, 2007; Le Be et al., 2007; Brown and Hestrin, 2009; Anderson et al., 2010; Dembrow et al., 2010; Sheets et al., 2011; Oswald et al., 2013). In the mPFC, CP and CA neurons have been denoted as PT and IT, respectively (Dembrow and Johnston, 2014). We therefore hypothesized that neurophysiological properties differed between CP and CA neurons in IL (**Figure 4A**). Whole-cell electrophysiology recordings in slice revealed that resting membrane potential (RMP) for L2 CA neurons was significantly hyperpolarized compared to both CP and L3/5 CA neurons (**Figure 4B**; **Table 1**). Input resistance of L3/5 CA neurons was significantly larger compared to CP and L2 CA neurons (**Figure 4C**; **Table 1**). This finding is consistent with L3/5 CA neurons being morphologically smaller than CP neurons (**Figure 3**). However, the soma diameter of reconstructed L2 CA neurons (15.8 ± 0.5 μm; *n* = 5), was not significantly different (Student’s unpaired *t*-test; *p* = 0.26) from L3/5 CA neurons (13.2 ± 1.9 μm; *n* = 9). Moreover, total dendrite length of L2 CA neurons (1.58 ± 0.4 mm) was statistically less (Student’s unpaired *t*-test; *p* < 0.05) than L3/5 CA neurons (3.0 ± 0.3 mm). Therefore, our data suggest that larger L3/5 CA input resistance is not related entirely to physical size.

**Table 1:** Intrinsic electrophysiological properties of IL neurons projecting to the PAG or BLA.

	CP (*n* = 26)	L3/5 CA (*n* = 20)	L2 CA (*n* = 10)	Significance
**Subthreshold**
Resting membrane potential (mV)	-68.5 ± 1.4	-66.1 ± 3.3	-76.0 ± 2.1	b, c
Input resistance (MΩ)	106 ± 4.4	196 ± 13	94.3 ± 8.7	a, c
Voltage sag (%)	8.8 ± 1.1	6.3 ± 1.4	-0.9 ± 0.4	b, c
Voltage overshoot (%)	13.0 ± 1.3	7.9 ± 1.1	0.7 ± 1.7	a, b, c
Resonant frequency (Hz)	2.2 ± 0.2	1.3 ± 0.3	0.7 ± 0.1	a, b
**Suprathreshold**
AP threshold (mV)	-38.4 ± 1.2	-32.5 ± 2.1	-38.4 ± 2.3	None
AP amplitude (mV)	74.6 ± 1.9	63.9 ± 2.8	68.0 ± 3.4	a
AP halfwidth (ms)	0.6 ± 0.03	0.67 ± 0.03	0.62 ± 0.03	None
Frequency current slope (Hz/pA)	0.15 ± 0.02	0.15 ± 0.01	0.17 ± 0.01	b
Fast SFA (3rd/5th)	0.93 ± 0.02	0.90 ± 0.01	0.89 ± 0.01	a
Slow SFA (2nd/Last)	0.76 ± 0.04	0.61 ± 0.03	0.69 ± 0.02	a
Fast after-hyperpolarization (mV)	-9.5 ± 0.8	-10.0 ± 1.6	-10.9 ± 1.3	None

CP, cortico-PAG neurons (18 animals); L3/5 CA, layer 3/5 CA neurons (14 animals); L2 CA, layer 2 CA neurons (seven animals). Data shown as mean ± SE of the mean. a, CP vs. L3/5 CA; b, CP vs. L2 CA; c, L3/5 CA vs. L2 CA. ANOVA (normally distributed data) or Kruskal–Wallace test (non-normally distributed data) followed by a Bonferroni *post hoc* analysis for multiple comparisons was used to determine statistical significance which was set at *p* < 0.05.

Voltage sag (**Figure 4D**; **Table 1**), which is an approximation of hyperpolarization-activated current (*I*_h_), has been shown to be substantially larger in PT neurons compared to IT neurons in motor cortex (Sheets et al., 2011; Oswald et al., 2013) and prefrontal cortex (Dembrow et al., 2010; Gee et al., 2012). In cortical pyramidal neurons, *I*_h_ stabilizes the RMP and influences the kinetics and propagation of synaptic responses (Spain et al., 1987; Nicoll et al., 1993; Williams and Stuart, 2000; Berger et al., 2001). L2 CA neurons had significantly less voltage sag than L3/5 CA and CP neurons (**Figure 4D**; **Table 1**). The absence of voltage sag for L2 CA neurons is consistent with an IT classification. Interestingly, average voltage sag for L3/5 CA neurons was similar to CP neurons (**Figure 4D**; **Table 1**). Application of the *I*_h_ blocker ZD7288 (10 μM) increased input resistance and eliminated voltage sag for both CP and L3/5 CA neurons (**Figures 4H–J**). This finding is not consistent with L3/5 CA neurons being an exclusive class of IT-like neurons.

Because neuronal resonance is influenced by *I*_h_ (Hutcheon and Yarom, 2000; Narayanan and Johnston, 2007; Shin et al., 2008; Dembrow et al., 2010; Sheets et al., 2011), we delivered sub-threshold chirp stimuli (frequency-swept sinusoids ranging linearly from 0 to 20 Hz over 20 s) and calculated impedance amplitude profiles (ZAPs) to estimate the frequency tuning and resonance properties of CP and CA neuronal subclasses in IL cortex (**Figure 4E**; **Table 1**). ZAPs of CP neurons exhibit broad peaks centered at ~2-3 Hz while CA neurons displayed more defined peaks at ~1 Hz (**Figure 4F**; **Table 1**). Overall, the resonant frequency of both L2 and L3/5 CA neurons was significantly less than CP neurons (**Figure 4G**; **Table 1**). Interestingly, these results did not correlate with our voltage sag findings suggesting that there are additional conductances affecting neuronal resonance in these subclasses of neurons. However, application of ZD7288 significantly reduced resonant frequency in both CP and resonating L5 CA neurons (**Figures 4K,L**). This once again suggests that L3/5 CA neurons are an intrinsically heterogeneous population of neurons with varying *I*_h_ expression.

**FIGURE 5:**
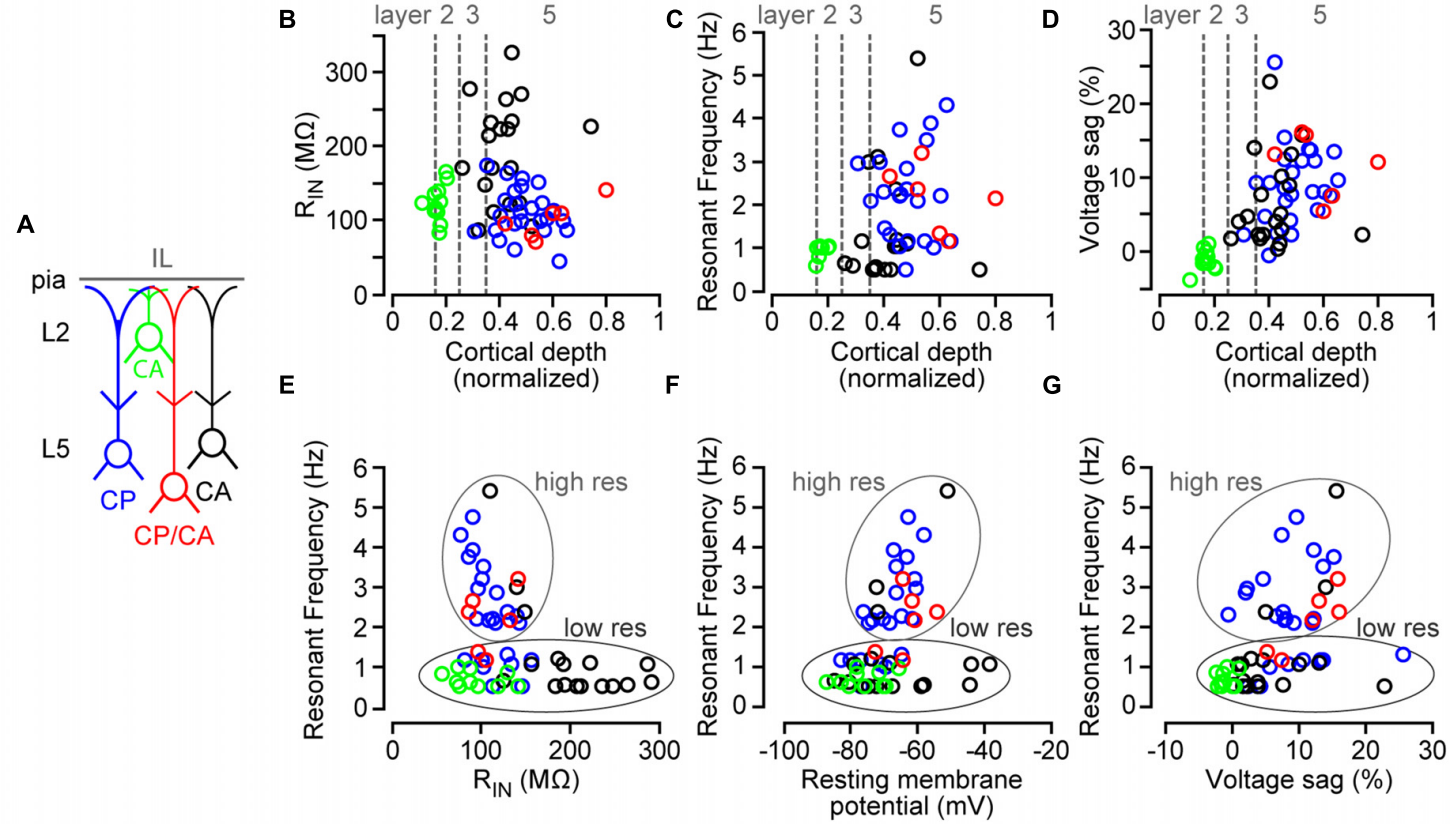
**Comparison of subthreshold properties as a function of cortical depth in IL and resonant frequency. (A)** Schematic showing the subclasses of neurons tested. **(B)** Input resistance, **(C)** resonant frequency, and **(D)** voltage sag as a function of normalized (pia = 0, white matter = 1) soma location for projection neuron classes. Mean normalized location of laminar borders are indicated with dashed gray lines. Plots of resonant frequency values versus **(E)** input resistance (R_IN_), **(F)** resting membrane potential, and **(G)** voltage sag percentage. High res = high resonators; low res = low resonators.

Looking closely at the subthreshold data, we observed a subset of L3/5 CA neurons with uncharacteristically low input resistance (**Figure 4C**). We hypothesized that a subset of these resonating L5 CA neurons also projected to the PAG as reported previously (Gabbott et al., 2005). To test this, we targeted and recorded from the small percentage of L5 IL neurons that were double labeled in following spectrally distinct retrograde tracers in the PAG and BLA (**Figures 2D,E**). For the four different classes of neurons (**Figure 5A**), we plotted input resistance, voltage sag, and neuronal resonance as a function of the precise position of the soma along the radial axis of the IL (**Figures 5B–D**). We found that, on average, CP/CA neurons (*n* = 6; six animals) were intrinsically similar to CP neurons showing low input resistance (101 ± 10 MΩ), large voltage sag (11.7 ± 1.8%), and high neuronal resonance (2.1 ± 0.3 Hz). This implies that the intrinsic variability observed for L3/5 CA neurons is due, in part, to having a subset of neurons that send collateral axonal projections to the PAG. Additionally, when comparing input resistance, RMP, and voltage sag with resonant frequency (**Figures 5E–G**), we found that high resonators (high res) consisted of a heterologous subpopulation which included CP, L3/5 CA, and CP/CA neurons. L2 CA neurons were exclusively low resonating (low res). However, a subpopulation of CP, L3/5 CA, and CP/CA neurons were also found to be low resonating (**Figures 5E–G**).

**FIGURE 6:**
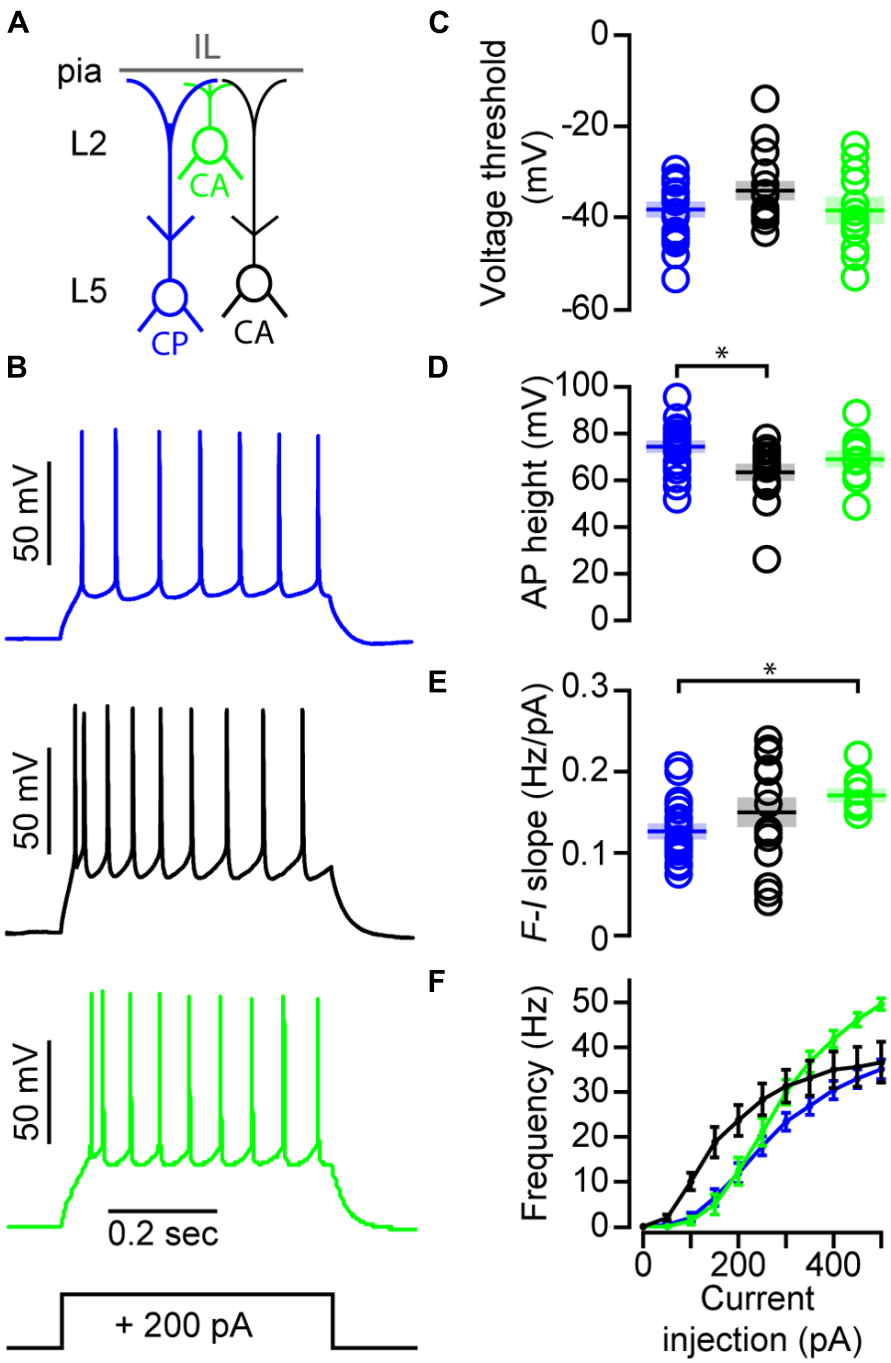
**Firing properties differ between CP and CA neurons in IL. (A)** Schematic showing the subclasses of neurons recorded. **(B)** Examples of action potential firing across projection class. **(C)** Voltage threshold, **(D)** AP height (*p < 0.05, Kruskal–Wallis test), **(E)** AP frequency-current slope (*p < 0.05, one-way ANOVA), and **(F)** AP frequency-current comparisons between CP (*n* = 26), L3/5 CA (*n* = 20), and L2 CA (*n* = 10) neurons. Shaded area = SEM.

Examination of suprathreshold properties (**Figures 6A,B**) revealed that voltage threshold for action potential (AP) firing was similar for CP, L3/5 CA, and L2 CA neurons (**Figure 6C**; **Table 1**). Slow spike frequency adaptation (SFA) during a train of APs, a characteristic of IT neurons, was present in all neuron classes (**Table 1**). CP and L3/5 CA neurons differed in AP height (**Figure 6D**; **Table 1**). Spike frequency-current slope (*F-I* slope) for L2 CA neurons was significantly greater than CP neurons but not L3/5 CA neurons (**Figure 6E**; **Table 1**). While the *F-I* slope was similar between CP and L3/5 CA neurons, frequency-current comparisons showed that L3/5 CA neurons fire APs much earlier that CP (**Figure 6F**). Frequency current comparisons also show that L2 CA neurons display a greater maximal firing rate compared to CP and L3/5 CA neurons (**Figure 6F**). Collectively, our data show that suprathreshold properties of CA and CP neurons in IL varied greatly.

### Projection Target is Correlated with Local Excitatory and Inhibitory Circuit Organization for CP and L3/5 CA Neurons

**FIGURE 7:**
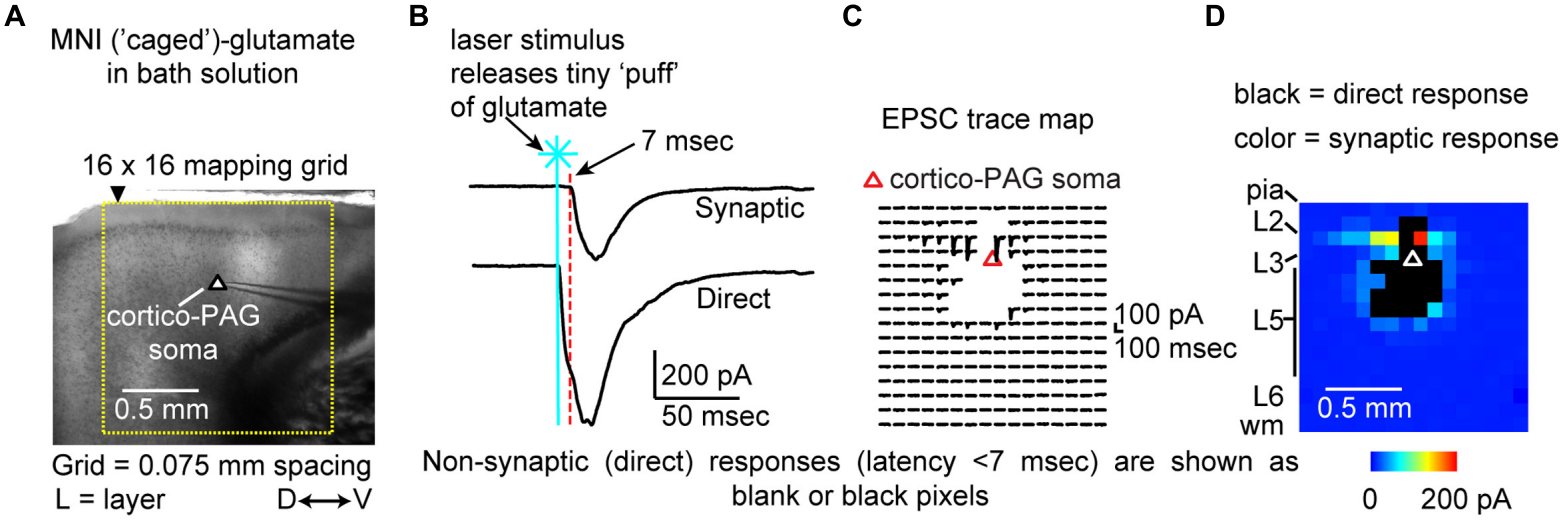
**Interpretation of local circuit mapping using laser-scanning photostimulation (LSPS). (A)** The soma of a retrogradely labeled neuron in acute brain slice is selected for whole-cell recording (D↔V: dorsal↔ventral). Release of caged-glutamate via UV laser creates a focal stimulus that is detected as a **(B)** post-synaptic (>7 ms latency) or direct event. UV laser stimulation is elicited in a 16 × 16 grid (75 μm spacing) tangent to the pia (i.e., midline) and horizontally centered on the soma of the recorded neuron (Δ). **(C)** An array of acquired traces is produced. Sites with direct responses are blanked. **(D)** A map of synaptic input is generated by color coding the array of synaptic input values at each site a pixelated image. Direct responses are presented as black pixels. Dorsal-ventral: D↔V.

Local circuit organization of pyramidal neurons in L5 of motor cortex differs based on their projection target (Anderson et al., 2010; Shepherd, 2014), and we hypothesized that this held true for CP and L3/5 CA neurons in IL. To test this, we mapped local excitatory and inhibitory circuits using focal activation of caged glutamate by laser scanning photostimulation (LSPS; **Figure 7**). Briefly, we obtained a whole-cell recording configuration for a retrogradely labeled cortical neuron in slice (**Figure 7A**). Next, glutamate was ‘uncaged’ via UV stimulation (20 mW, 1 ms) in a 16 × 16 grid (75 μm spacing) tangent with the pia and centered horizontally with the neuron soma. Responses were recorded in voltage-clamp mode (**Figure 7B**) to produce a trace map (**Figure 7C**) of input from local presynaptic locations which we converted to color maps for visualization (**Figure 7D**).

**FIGURE 8:**
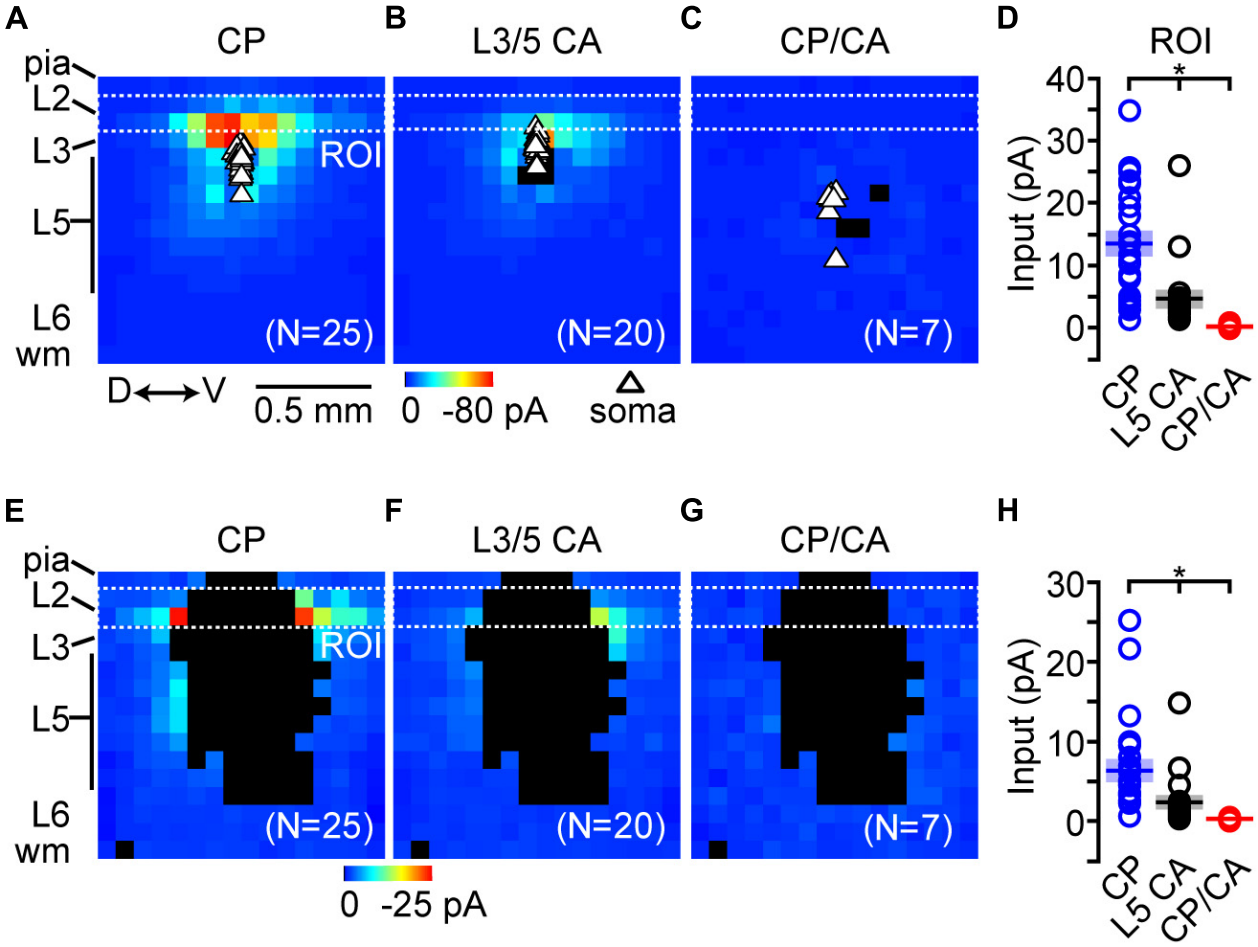
**Local excitatory circuit mapping of projectionally defined IL neurons.** Average excitatory local circuit maps for **(A)** CP, **(B)** L3/5 CA neurons, and **(C)** CP/CA neurons (D↔V: dorsal↔ventral, ROI: region of interest). **(D)** Mean (±SEM) of excitatory input originating in L2 (^∗^*p* < 0.05, Kruskal–Wallis test). Average local circuit maps following the application of a direct mask for **(E)** CP, **(F)** L3/5 CA, and **(G)** CP/CA neurons. **(H)** Statistical significance of input originating in L2 (ROI) remained unchanged following application of the direct masks (^∗^*p* < 0.05, Kruskal–Wallis test). Shaded area = SEM.

Compilation of excitatory synaptic input maps (*n* = 25; 16 animals) showed that CP neurons receive strong local input from L2 (**Figure 8A**). Comparatively, L3/5 CA neurons (*n* = 20; 10 animals) received significantly less local excitatory input from L2 (**Figures 8B,D**). Mapping experiments on CP/CA neurons (**Figure 8C**) showed that excitatory inputs originating in L2 were significantly smaller compared to CP and L3/5 CA neurons (**Figure 8D**). A caveat here is that L3/5 CA neurons are distributed close to L2 increasing the probability of direct stimulation to their proximal dendrites via glutamate uncaging. This direct stimulation of dendrites potentially masks synaptic inputs thereby dampening the potential impact of L2 inputs to CA neurons. To address this issue, we created a mask which accounted for all locations where glutamate uncaging directly stimulated the dendrites of the recorded neuron for all groups. After applying the direct mask, the average L2 input for both groups decreased, but the significant difference between CP, L3/5 CA, and CP/CA neurons remained unchanged (**Figures 8E–H**). Previous findings in motor cortex show that excitatory input from L2 decreases with cortical depth of the soma (Anderson et al., 2010). Most mapping experiments were targeted to labeled somas close to the L3/L5 border to improve the odds of receiving strong L2 input. As mentioned previously, CP/CA neurons were found primarily at deeper cortical depths which made robust L2 input unlikely.

**FIGURE 9:**
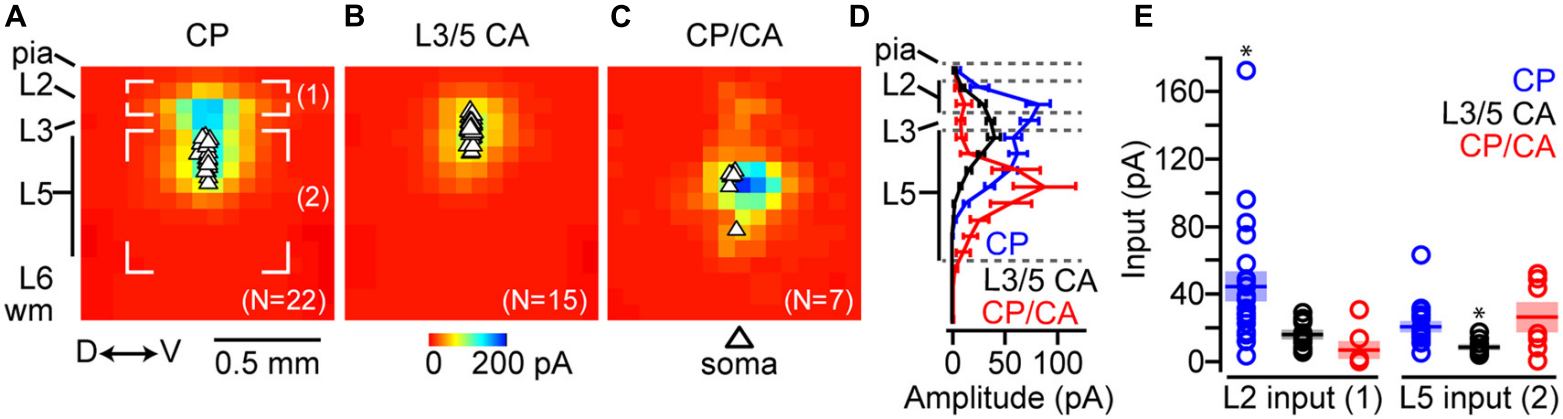
**Local inhibitory circuitry depends on specific projection target.** Average local inhibitory circuit maps for **(A)** CP, **(B)** L3/5 CA neurons, and **(C)** CP/CA neurons (D↔V: dorsal↔ventral). **(D)** Laminar profile of inhibitory input following glutamate uncaging (75 μm bins, mean ± SEM; dashed lines = approximate laminar borders). **(E)** Mean (±SEM) of inhibitory responses (^∗^*p* < 0.05, Kruskal–Wallis test) following excitation of L2 (ROI 1) and L5 (ROI 2). Shaded area = SEM.

We also mapped local sources of inhibitory inputs using a Cs^+^-based internal solution (voltage-gated K^+^-channel inhibition) that included QX-314 (voltage-gated Na^+^ channel inhibition). This solution allowed us to hold recorded neurons at the reversal potential for glutamatergic current (~0 – +10 mV), and therefore detect local inhibitory inputs elicited by glutamate uncaging. Here, direct excitatory stimulation of proximal dendrites does not affect input maps because we are holding at the reversal potential for glutamatergic current. We recorded inhibitory input maps from CP neurons (*n* = 22; 15 animals), L3/5 CA neurons (*n* = 15; seven animals), and CP/CA neurons (*n* = 6; six animals) following excitation of L2 (region of interest 1) and L5 (region of interest 2) and performed a region-of-interest analysis similar to the excitatory maps (**Figures 9A–C**). Inhibitory responses driven by L2 excitation are most likely caused by disynaptic feedforward inhibition as previously observed in motor cortex (Apicella et al., 2012). Inhibitory responses driven by L5 excitation are most likely a combination of direct excitation of L5 inhibitory neurons and disynaptic feedforward inhibition within L5. When we pooled the inhibitory maps for each group, row analysis of inputs showed that CP and L3/5 CA neurons were broadly inhibited following excitation of L2, L3, and L5 (**Figure 9D**). Inhibition detected in CP/CA neurons was robust following excitation deeper in L5 (**Figure 9D**). Comparatively, CP neurons received the strongest inhibitory input following excitation of L2 (**Figure 9E**). This disparity may be explained by previous findings showing that local connectivity of parvalbumin-positive fast spiking (FS) interneurons is stronger with PT versus IT neurons in mPFC (Lee et al., 2014). Broad activation of L5 resulted in significantly larger inhibitory inputs to CP and CP/CA neurons compared to L3/5 CA neurons (**Figure 9E**). Because inhibition driven by L5 excitation was mainly perisomatic, location of inputs varied between neuronal groups due to different soma locations in L5.

## Discussion

We show that cortical neurons projecting to two different subcortical areas involved in fear mechanisms, the PAG and the BLA, are uniquely distributed in the IL and have significant differences in morphology, intrinsic physiology, and local circuitry organization. These differences provide insight into the cortical regulation of fear perception and corresponding autonomic responses. Here, retrograde labeling experiments show that pyramidal neurons in L5 of the IL project to the PAG, a structure involved in panic behavior and emotional coping pathways (Keay and Bandler, 2001; Graeff and Del-Ben, 2008). Specifically, labeled neurons projecting to ventral PAG (vPAG) were distributed in L5 of IL. This distribution of CP neurons has mechanistic implications for why IL stimulation contributes to fear extinction behavior (Vidal-Gonzalez et al., 2006). This is because activating the vPAG drives quiescence and decreased blood pressure (Bandler and Shipley, 1994; Bandler et al., 2000). Therefore, descending IL output pathways targeting the vPAG may be a significant contributor to the autonomic response associated with fear extinction.

Reciprocal connectivity between the mPFC and the BLA is also crucial for fear conditioning (see review Marek et al., 2013). Here we show that a significant portion of mPFC output to the BLA consists of pyramidal neurons distributed in L2, L3, and L5 of IL. Anterograde tracing in rat has shown that IL neurons project not only to the BLA, but to the GABAergic central nucleus and intercalated nuclei (ITC) of the amygdala (McDonald et al., 1996; Berretta et al., 2005; Pinard et al., 2012). In fear extinction, increased activation of the ITC drives inhibition of output neurons in the central amygdala (Amano et al., 2010). A key target of these central amygdala neurons is the PAG (LeDoux et al., 1988). This suggests that the IL can affect PAG activity directly and indirectly through the circuitry of the amygdala. A critical remaining question is whether the laminar location of CA neurons (i.e., L2 vs. L3/5) in IL determines their specific target within the amygdala.

Cortico-PAG and CA neurons in the mPFC can be classified as PT and IT, respectively (Dembrow and Johnston, 2014). PT and IT neurons in frontal cortex (Morishima and Kawaguchi, 2006; Otsuka and Kawaguchi, 2008; Dembrow et al., 2010; Hirai et al., 2012) and other areas of cortex (Kasper et al., 1994; Hattox and Nelson, 2007; Le Be et al., 2007; Sheets et al., 2011; Oswald et al., 2013) can be distinguished by morphology, intrinsic electrophysiological properties, and local circuit connectivity. Morphologically, PT neurons in rat frontal cortex have been shown to have larger apical dendritic length and deeper apical bifurcations compared to IT neurons (Morishima and Kawaguchi, 2006; Dembrow et al., 2010). However, these studies found no difference in soma size between PT and IT neurons. We found that somas of CP neurons in mouse IL were significantly larger than neighboring L3/5 CA neurons. Additionally, apical tuft width, and both the apical and basal dendritic length of CP neurons were significantly larger that L3/5 CA neurons. This observed difference in soma size and dendritic structure between CP and L3/5 CA neurons suggests that they have distinct integrative capabilities when receiving both local and long-range inputs.

Neuronal resonance and large voltage sag caused by h-current (*I*_h_) are characteristics of PT neurons in the mPFC (Dembrow et al., 2010) and motor cortex (Sheets et al., 2011). For instance, L5 corticopontine neurons (PT) express significantly higher resonance and voltage sag compared to neighboring commissural neurons (IT) in the rat mPFC (Dembrow et al., 2010). Collectively, we found that CP, CA, and CP/CA neurons could be broadly classified as high resonators and low resonators (**Figure 5**). Only L2 CA neurons were exclusively low resonators that were hyperpolarized at resting and void of voltage sag (**Figure 4**). These characteristics place L2 CA neurons firmly in the IT categorization. For L3/5 in IL, low resonating and high resonating categories were made up of heterogeneous populations of CP, CA, and CP/CA neurons. This indicates that in L3/5 of IL, projection target does not exclusively correlate with levels of neuronal resonance as seen previously in the rat mPFC (Dembrow et al., 2010) and mouse motor cortex (Sheets et al., 2011). Variability in input resistance and voltage sag was also observed for these L3/5 projection classes of neurons. This suggests that L3/5 neurons in IL cannot be considered exclusively PT and IT based on projection target. Nonetheless, we observed populations of L3/5 CA neurons that did not resonate and expressed high input resistance (**Figure 5E**). It could be argued that this a class of pure CA neurons that project only to the amygdala while the other class of resonating, low-impedance L3/5 CA neurons represent a subpopulation that send axon collaterals to other PT subcortical targets such as the pons or thalamus. Additionally, we found non-resonating CP neurons with negligible voltage sag. Are these CP neurons with axon collaterals to IT targets? We know there is a subpopulation of CP neurons that also send projections to the BLA (**Figure 2**). However, only two out of six CP/CA neurons were low resonators (**Figure 5**). Therefore, the low resonance observed in a subset of CP neurons cannot be exclusively attributed to additional projections to IT targets.

The variation of *I*_h_ in IL neurons studied here may be relevant to how neuromodulatory pathways affect cortical activity. For instance, decrease of *I*_h_ via adrenergic stimulation in brain slices of ferret prefrontal cortex causes an increase in overall activity, presumably due to enhanced synaptic integration (Wang et al., 2007). In IL, noradrenergic and dopaminergic signaling, which can also affect *I*_h_ levels, are essential for fear extinction (Mueller et al., 2008, 2010). Therefore, specific components of fear extinction may be driven by modulatory inputs that regulate IL output by altering levels of *I*_h_ in a subset of CP, L3/5 CA, and CP/CA neurons. Our findings also suggest that due to lack of *I*_h_, L2 CA neurons would not be sensitive to this neuromodulation.

Communication between the PAG and amygdala is instrumental in fear learning (McNally et al., 2011). Pharmacologic inhibition of the PAG reduces the amygdalar response to aversive stimuli thereby impairing fear conditioning (Johansen et al., 2010). More specifically, antagonism of opioid receptors in vPAG reduces the development of fear extinction (McNally et al., 2004; Parsons et al., 2010). Based on our findings and others (Quirk et al., 2000, 2003; Gabbott et al., 2005), the local circuitry within IL is essential in top–down control of both the PAG and BLA. Therefore, connectivity between CP and CA neurons in IL is likely a critical component of top–down control of fear extinction by IL (Vidal-Gonzalez et al., 2006; Sierra-Mercado et al., 2011). Our local circuit mapping in IL revealed that CP neurons receive strong excitatory input from L2. Pair-recording studies in the motor and frontal cortex have shown local connections of IT neurons onto PT neurons (Morishima and Kawaguchi, 2006; Kiritani et al., 2012) including L2–L5 connections (Hirai et al., 2012). Thus, the robust expression of CA neurons in L2 of IL suggests that CP activity, at least in part, is driven by local descending L2 CA input. Circuit mapping did not reveal a strong local excitatory source of input to CP/CA neurons. This implies that activity of CP/CA neurons is mainly driven by long-range presynaptic inputs which potentially originate from the BLA, thalamus, and contralateral IL. Future experiments involving paired recordings and/or optogenetic techniques testing connectivity between CP and CA neurons and long-range inputs to IL are necessary for confirming this level of organization.

Glutamate uncaging in IL also produced inhibitory inputs to CP and L3/5 CA neurons likely caused by both disynaptic feedforward inhibition and direct stimulation of inhibitory interneurons. Excitation of L2 produced greater interlaminar inhibition of L5 CP neurons compared to L3/5 CA neurons. These results suggest that low-threshold spiking interneurons in L5, which are excited by L2 pyramidal neurons (Otsuka and Kawaguchi, 2009; Apicella et al., 2012), connect more robustly with CP neurons than CA neurons. Additionally, perisomatic inhibition was greater for CP and CP/CA neurons compared to L3/5 CA neurons. The disparity may be explained by findings showing parvalbumin-positive FS interneurons connect more strongly with PT versus IT neurons in mPFC (Lee et al., 2014). Weak excitatory input to CP/CA neurons suggests that the measured inhibitory inputs are a result of direct stimulation of local IL interneurons. It will be interesting to identify intralaminar connection specificity between inhibitory interneurons, CP, CA, and CP/CA neurons in IL and how it affects the dynamics of mPFC output in heightened fear and pain states.

Our findings describe a framework for the organization, intrinsic properties, and local circuitry of projectionally defined IL neurons targeting subcortical structures crucial in fear learning. Integration of converging local and long-range inputs to CP and CA neurons is undoubtedly critical for controlling the IL output necessary for refining fear extinction. Importantly, increased burst firing is observed in BLA neurons projecting to PL and IL following fear conditioning and fear extinction, respectively (Senn et al., 2014). Results from this study suggest that this change in AP dynamics elicits shifts in the excitation and inhibition circuitry controlling CP and CA output from the IL. Continued effort to dissect both the local and long-range networks altering CP and CA activity in IL is critical for understanding the consolidation of fear expression and extinction memories.

## Author Contributions

AF and PS performed all experiments and analysis, with contributions from HY and SD. PS interpreted the data and wrote the paper.

## Conflict of Interest Statement

The authors declare that the research was conducted in the absence of any commercial or financial relationships that could be construed as a potential conflict of interest.

## Abbreviations

BLA: basolateral nuclei of the amygdala
CA: cortico-BLA
CP: cortico-PAG
glu-LSPS: glutamate uncaging via laser scanning photostimulation
IL: infralimbic cortex
L2: layer 2
L3: layer 3
L5: layer 5
L3/5: layer 3 and layer 5
mPFC: medial prefrontal cortex
PAG: periaqueductal gray.
